# Universal dynamics of mitochondrial networks: a finite-size scaling analysis

**DOI:** 10.1038/s41598-022-14946-9

**Published:** 2022-10-12

**Authors:** Nahuel Zamponi, Emiliano Zamponi, Sergio A. Cannas, Dante R. Chialvo

**Affiliations:** 1 Division of Hematology and Medical Oncology, Department of Medicine, Weill Cornell Medicine, 1300 York Avenue, New York, NY 10065 USA; 2 Department of Molecular, Cellular, and Developmental Biology, University of Colorado-Boulder, Boulder, CO 80309 USA; 3 Present Address: Department of Neuroscience, Mortimer B. Zuckerman Mind Brain Behavior Institute, Columbia University, New York, NY 10027 USA; 4 Facultad de Matemática Astronomía Física y Computación, Universidad Nacional de Córdoba, Instituto de Física Enrique Gaviola (IFEG-CONICET), Ciudad Universitaria, 5000 Córdoba, Argentina; 5 Consejo Nacional de Investigaciones Científicas y Técnicas (CONICET), Godoy Cruz 2290, 1425 Buenos Aires, Argentina; 6 Instituto de Ciencias Físicas (ICIFI-CONICET), Center for Complex Systems and Brain Sciences (CEMSC3), Escuela de Ciencia y Tecnología, Universidad Nacional de Gral. San Martín, Campus Miguelete, 25 de Mayo y Francia, 1650 San Martín, Buenos Aires, Argentina

**Keywords:** Complexity, Criticality, Mitochondria

## Abstract

Evidence from models and experiments suggests that the networked structure observed in mitochondria emerges at the critical point of a phase transition controlled by fission and fusion rates. If mitochondria are poised at criticality, the relevant network quantities should scale with the system’s size. However, whether or not the expected finite-size effects take place has not been demonstrated yet. Here, we first provide a theoretical framework to interpret the scaling behavior of mitochondrial network quantities by analyzing two conceptually different models of mitochondrial dynamics. Then, we perform a finite-size scaling analysis of real mitochondrial networks extracted from microscopy images and obtain scaling exponents comparable with critical exponents from models and theory. Overall, we provide a universal description of the structural phase transition in mammalian mitochondria.

## Introduction

The arise of mitochondria constitutes a milestone in the evolution of eukaryotes. Their incorporation into the proto-eukaryotic cell made possible a significant increase in genome complexity by allowing the cell to afford the energetic cost of a bigger proteome^1^. In most extant eukaryotes, mitochondria have evolved to become crucial organelles in energy metabolism, anabolism, and essential regulators of cell death^2–5^. Moreover, most mitochondrial genes were transferred to the nuclear genome, facilitating the emergence of complex regulatory networks that constantly match mitochondrial activity with the metabolic demands of the cell while maintaining the organelle’s autonomy^6–8^.

**Figure 1:**
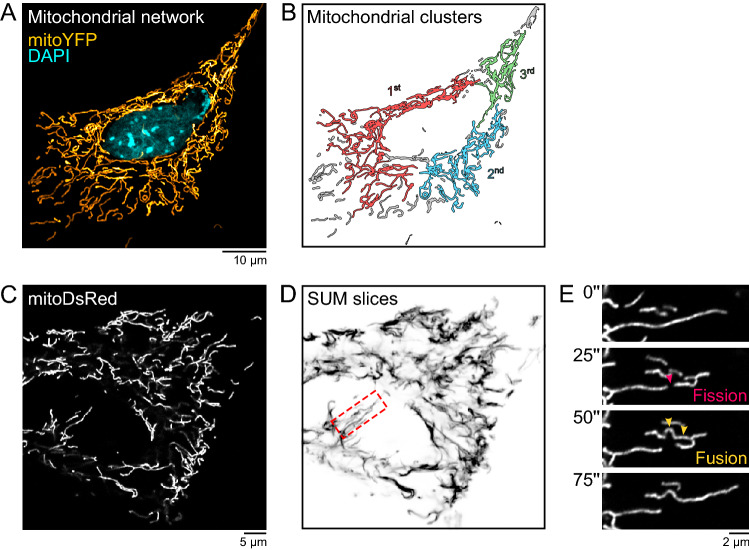
Mitochondrial network structure and dynamics. (**A**) Mitochondrial network of a mouse embryonic fibroblast (MEF) expressing a mitochondria-targeted yellow fluorescent protein (mitoYFP). (**B**) Segmentation and identification of the most relevant clusters in the network shown in (**A**). (**C**) Live-imaging of a MEF expressing a mitochondria-targeted red fluorescent protein (mitoDSRed). (**D**) Projection of different time frames revealing regions in the network with slow (dark) and fast (light) mitochondrial dynamics. (**E**) Zoom-in of the inset in (**D**) highlighting specific fission and fusion events occurring in the network.

Inside cells, mitochondria arrange in intricate networks composed of clusters of different sizes (Fig. 1A and B)^9,10^. The size distribution of these clusters differs across cell types and is associated with the energetic state of each cell^11,12^. Mitochondrial clusters constantly break down and fuse through mitochondrial fission and fusion processes, favoring the assembly of oxidative phosphorylation protein complexes with the correct stoichiometry and maintaining the integrity of the mitochondrial DNA (Fig. 1C, D, and E)^13–20^. In addition, fission and fusion ensure mitochondrial network homeostasis by allowing damaged mitochondria to be either fused to healthy mitochondria and rescued by content mixing or, if the damage is irreversible, excised from the rest of the network and recycled in a process called mitophagy^4,21–24^.

In many mammalian cells, the size distribution of mitochondrial clusters is scale-free, suggesting that mitochondrial dynamics are poised at the critical point of a phase transition since the absence of any characteristic scale in the system’s variables is known to be a hallmark of criticality^25^. Moreover, the spontaneous emergence of a coherent ensemble in the form of a giant mitochondrial cluster from fission and fusion dynamics occurring at the molecular level traces back to critical phenomena because it resembles the type of behavior found in critical dynamics in models and theory^26–33^. Several results support the notion that critical phenomena are relevant to mitochondrial structure and function^34–40^. Regardless of the importance of these findings, evidence demonstrating that the expected finite-size scaling behavior holds in mitochondria has not been provided yet.

The theory states that the correlation length diverges at the critical point, and the system quantities become scale invariant^25,32,41,42^. For finite-size systems, this implies that the value of such quantities at the critical point will grow monotonically with the system’s size. Let us consider the size of the second largest cluster, which in percolating systems reaches its maximum size at the critical point^43^. At criticality, the prediction is that the size of the second largest cluster will increase with the size of the system following a specific scaling law. The tools for deriving all the relevant scaling laws and their associated critical exponents are known collectively as finite-size scaling analysis. Systems can be classified according to their critical exponents in different universality classes, i.e., sets of mathematical models that behave similarly at a large scale^44,45^.

We hypothesize that the fission-fusion dynamics that give rise to the characteristic networked structure found in mitochondria operate near criticality. In this context, the lack of a direct measurement of the expected scaling behavior is a major missing piece of evidence. Here we fill this gap by demonstrating scaling behavior in mitochondrial networks: 1) We identify the control parameter and determine how the relevant network quantities scale with the network’s size at the critical point in two different models of generic mitochondrial dynamics. Furthermore, the exponents obtained from these simulations let us identify the universality class the models belong to. 2) Utilizing a similar strategy, we characterize the scaling behavior of the same network quantities in real mitochondrial networks obtained from microscopy images. 3) We compare the exponents obtained from simulations and real networks with the theoretical ones and demonstrate that mitochondrial dynamics are critical and that they seem to belong to the 2D percolation universality class.

The paper is organized as follows. In the next section, the two models used in the study are introduced together with the approach utilized to extract network data from images of mitochondria. We then define the quantities measured from networks and the scaling relationships employed in the finite-size scaling analysis. The results section contains a detailed characterization of the models, including the behavior of the relevant quantities as a function of the control parameter and the finite-size scaling analysis. A similar description is provided for real mitochondrial networks extracted from microscopy images. The paper closes with a discussion on the relevance of the present results to understanding mitochondrial dynamics. Further details about the methods can be found in a dedicated section.

## Simulated *vs.* real mitochondrial networks

### Models of mitochondrial dynamics

**Figure 2:**
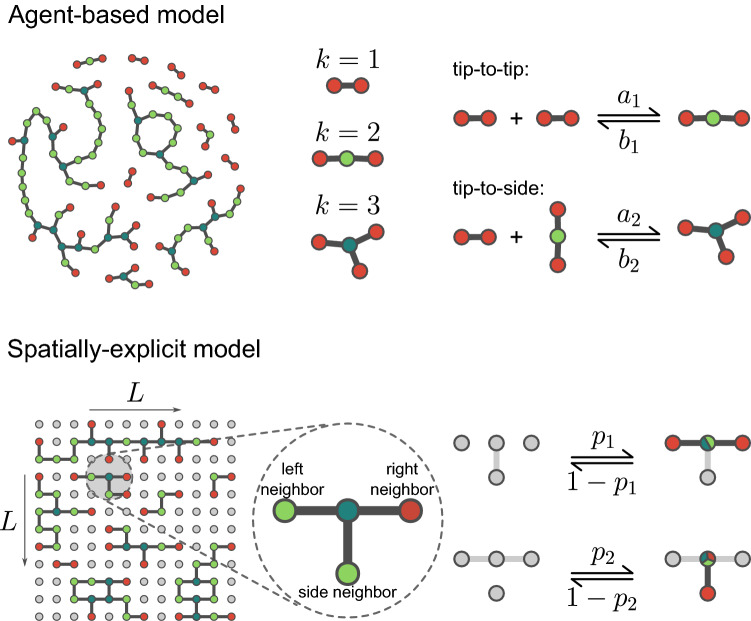
The two models of mitochondrial network dynamics used in this work. In the agent-based model, network nodes do not have explicit spatial coordinates. The final topology of the network emerges from the iteration of two types of events: tip-to-tip events, in which two $k=1$ units are merged into a $k=2$ unit (or vice versa), and tip-to-side events, in which a $k=1$ unit and a $k=2$ unit are merged into a $k=3$ unit (or vice versa). In the spatially-explicit model, the network nodes are embedded in a 2-dimensional lattice with predetermined nearest neighborhood interactions. Interactions are anisotropic: a bond is established between a node and its left and right nearest neighbors with probability $p_1$ (or destroyed with probability $1 - p_1$) and with its side nearest neighbor with probability $p_2$ (or destroyed with probability $1 - p_2$).

The only agent-based model of the mitochondrial network available was introduced recently^38^. In their original contribution, the authors demonstrated that the model exhibits a percolation phase transition as a function of one of the fission/fusion parameters and that the network configurations emerging at the critical point were consistent with the ones from real mitochondrial networks. This model, termed *agent-based (AB) model* (Fig. 2, top), assumes three types of nodes, namely, free ends of mitochondrial segments ($k = 1$), bulk sites ($k=2$) and branching points ($k=3$). Links between nodes (edges) represent minimal mitochondrial fragments and define the scale of the network. The total number of edges $N_e$ is kept fixed throughout the simulation. Model dynamics evolve through tip-to-tip and tip-to-side fission/fusion reactions of the type

1 $$\begin{aligned} 2\,X_1&\rightleftharpoons X_2, \end{aligned}$$

2 $$\begin{aligned} X_1 + X_2&\rightleftharpoons X_3, \end{aligned}$$

where $X_i$ ($i=1,2,3$) corresponds to nodes with degree *i*. Tip-to-tip reactions happen with association (dissociation) rate $a_1$ ($b_1$) between a random pair of nodes with degree $k = 1$ (association) or a random site with degree $k = 2$ (dissociation). Tip-to-side reactions happen with association (dissociation) rate $a_2$ ($b_2$) between a random pair of nodes with degrees $k = 1$ and $k = 2$ (association) or for a random site with degree $k = 3$ (dissociation)^46^. Following *Sukhorukov et al.*, we take into account that only one type of fission is found experimentally and assume $b_2=(3/2)b_1$, and varied the relative rates $c_i=a_i/b_i$^38,47^. Notice that network edges are the model’s minimal (indivisible) elements, analogous to the smallest mitochondrial fragment found in nature, and fusion and fission processes correspond to network nodes’ transformations, analogous to the cellular machinery responsible for fusing and or excising mitochondrial segments. The AB model constitutes a mean-field approximation and therefore does not account for the positions of the nodes in space nor contains information on the distance between mitochondrial clusters (i.e., it has infinite spatial dimensions). However, mitochondria are embedded in the cellular volume, which in most “flat” cells can be mapped to a 2-dimensional space (Supplementary Fig. 1)^48–57^. Therefore, to include this information in our simulations, we derived an additional model, termed *spatially-explicit (SE) model* (Fig. 2, bottom), inspired by the spatial orientation of mitochondrial fragments during fission and fusion events^58–60^. In this model, nodes are embedded in a 2D lattice whose positions are fixed. In contrast to the mean-field equations ruling the evolution in the AB model, the SE model evolution is dictated by probabilities concerning the local connectivity of randomly selected nodes. Furthermore, the SE model assumes two types of neighbors (that imply two different types of bonds): near neighbors, comprising the two nearest nodes within the same lattice row, and the side neighbor, referring to the nearest neighbor within the same lattice column. At any given time, a bond between a random node and its left and right neighbors is established with probability $p_1$ (or destroyed with probability $1-p_1$). Similarly, a bond between the same random node and its side neighbor is established with probability $p_2$ (or destroyed with probability $1-p_2$). Using these two models, we can determine the upper and lower boundary conditions for the universal behavior of real mitochondria, represented by network dynamics in spaces with infinite and two dimensions, respectively.

### Real mitochondrial networks

We obtained data from confocal images of MEFs infected with a lentivirus carrying a mitochondria-targeted yellow fluorescent protein (mitoYFP). Images were processed as described elsewhere to obtain pixel masses of individual mitochondrial clusters within each cell^34^. In addition, different intensity thresholds were used during image processing to account for the heterogeneity in mitoYFP distribution and out-of-plane illumination (see “Methods”).

## Phase transitions

We hypothesize that the complex topology of mitochondrial networks emerges from the interaction of small mitochondria constituents at the critical point of a phase transition^61^. To simulate this, we identified the region in the parameters space of the models where the phase transition occurs by measuring the following quantities^62^: the average fraction of nodes in the largest cluster $\langle N_g/N \rangle$, the average number of nodes in the second largest cluster $\langle N_2 \rangle$, the average cluster size $\langle s \rangle$ and the complementary cumulative distribution function (CCDF) associated to $n_s$, namely,

3 $$\begin{aligned} N_{c}(s) = \sum ^{'}_{s' \ge s} n_{s}(s'), \end{aligned}$$

where the primed sum excludes the giant cluster.

$\langle N_g/N \rangle$ is the order parameter of the transition since it informs when a giant cluster emerges in the network (i.e., when the transition from the disordered phase to the ordered phase takes place). In addition, the system’s susceptibility is expected to be maximal at the critical point, reflecting the presence of long-range correlated fluctuations. Here, $\langle s \rangle$ is our proxy for the system’s susceptibility and is calculated using the expression from classical percolation theory^62–65^, namely if $N_s$ is the number of clusters of size *s* and $n_s = N_s/N$, then

4 $$\begin{aligned} \langle s \rangle = \frac{\sum ^{'}_s s^2 n_s}{\sum ^{'}_s s n_s}, \end{aligned}$$

where the primed sums exclude the largest cluster in the network.

## Finite-size scaling and universality

The emergence of a coherent ensemble in the form of a dynamic giant cluster resembles the type of collective behavior characterizing many physical and biological systems in which correlations are amplified in the vicinity of the critical point^66,67^. However, the critical point is only sharply defined in the thermodynamic limit, away from which the (effective) critical value of the control parameter depends on the system’s size. Simply put, a value of the control parameter that is critical for a system of size *N* will be off for a larger system^42^. Consequently, quantities like $\langle s \rangle$ and $\langle N_2 \rangle$ are expected to exhibit size-dependent maxima following well defined scaling laws^43,62^

5 $$\begin{aligned}&\langle s \rangle |_{max} \sim N^{\gamma / \nu d}, \end{aligned}$$

6 $$\begin{aligned}&\langle N_{2} \rangle |_{max}\sim N^{d_{f}/d}. \end{aligned}$$

Here, $\gamma$ and $\nu$ are the standard susceptibility and the correlation length critical exponents, respectively, *d* is the effective dimension of the system (equal to the spatial dimension *D* if $D < d_{c}$ (the upper critical dimension) or to $d_c$, otherwise) and $d_f$ is the fractal dimension of the percolating cluster. The specific values of these parameters determine the universality class to which the system under scrutiny belongs^68^.

In addition, the cluster size distribution is expected to follow a power-law with a size-dependent exponential cutoff of the form

7 $$\begin{aligned} N_c(s) \sim s^{-(\tau - 1)} \ e^{-s/s^*}, \end{aligned}$$

where $\tau$ corresponds to the Fisher exponent and the term $e^{-s/s^*}$ corresponds to the exponential cutoff with $s^* \propto N_2$^62,69^.

In the following sections we will utilize these scaling exponents to determine the universality class to which mitochondrial dynamics belong.

## Results

### Critical exponents of the AB model deviate from mean-field percolation universality class

**Figure 3:**
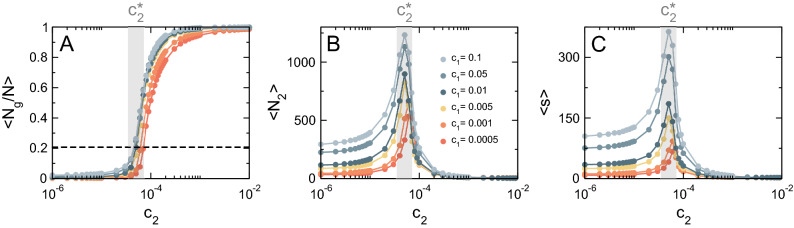
Phase transition in the AB model. (**A**) Order parameter $\langle N_g/N \rangle$ as a function of the control parameter $c_2$ for a system of size $N_e = 1.5 \times 10^4$ and different values of $c_1$. Shaded region depicts the pseudo–critical value $c^{*}_2$ for $c_1 = 0.01$. Dashed line denotes the proportion of nodes belonging to the largest cluster at the critical point for $c_1 = 0.01$. (**B**) Size of the second largest cluster $\langle N_2 \rangle$ as a function of $c_2$. (**C**) Susceptibility $\langle s \rangle$ as a function of $c_2$.

Figure 3 illustrates the typical behavior of the AB model as a function of $c_2$^34,38^. The system undergoes a second order phase transition at a pseudo–critical value of the control parameter $c_2^*$, reflected in a steep change in the normalized size of the largest cluster (order parameter), as shown in Fig. 3A^43^. Concomitantly with the emergence of a giant cluster, the size of the second largest cluster $\langle N_2 \rangle$ peaks at $c_2^*$ (Fig. 3B). Equally distinct changes are also demonstrated for the system’s susceptibility computed as $\langle s \rangle$ (Fig. 3C). The peak in $\langle s \rangle$ implies that fluctuations are correlated over longer distances at the critical point. Gray regions highlight that the emergence of the largest cluster and the peaks in $\langle N_2 \rangle$ and $\langle s \rangle$ coincide.

**Figure 4:**
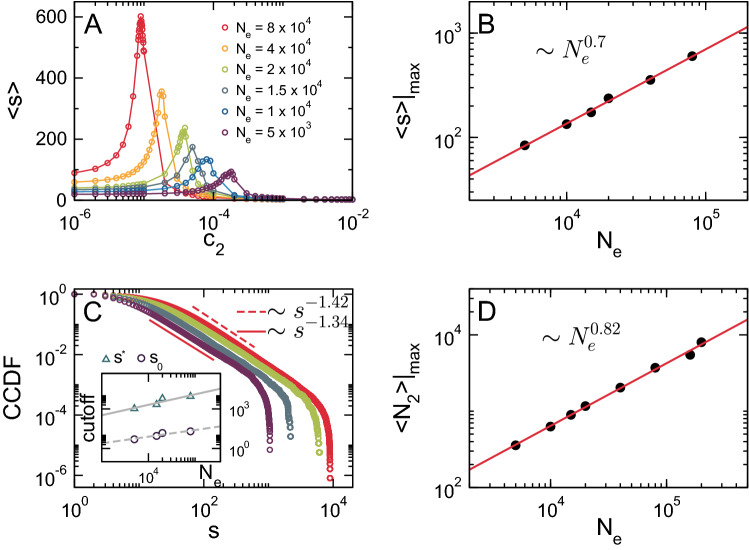
Finite-size scaling analysis of the AB model. (**A**) Susceptibility $\langle s \rangle$ as a function of $c_2$ for different system sizes ($c_1 = 0.01$). $\langle s \rangle$ exhibits a size-dependent maximum at a pseudo–critical value $c_2^*(N_e)$. (**B**) Log-log plot of $\langle s \rangle |_{max}$ as a function of $N_e$, where the solid red line is a power-law fitting with exponent $0.7\,\pm \,0.01$. (**C**) The CCDF of the cluster size distribution at $c_2^*(N_e)$ for different system sizes, where dashed and solid lines correspond to power-law fittings of the central part of the distribution with exponents $-1.42$ $(N_e = 8 \times 10^{4})$ and $-1.34$ $(N_e = 5 \times 10^{3})$, respectively. The inset shows the scaling of small-size ($s_0$) and large-size ($s^*$) cutoffs of the distribution as a function of $N_e$, where solid and dashed lines correspond to power-law fits with exponents $0.8\,\pm \,0.2$ and $0.5\,\pm \,0.1$, respectively. (**D**) Log-log plot of $\langle N_2 \rangle |_{max}$ as a function of $N_e$, where the solid red line is a power-law fitting with exponent $0.82\,\pm \,0.01$.

At finite size, the effective critical value of the control parameter depends on the system’s size^42^. Moreover, as stated in the previous section, quantities such as $\langle s \rangle$ and $N_2$ are expected to show size-dependent maxima. To characterize the universal properties of the AB model, we performed a finite-size scaling analysis of relevant network quantities, namely $N_c(s)$, $\langle s \rangle$ and $\langle N_2 \rangle$. As shown in Fig. 4A, the system’s susceptibility exhibits a series of maxima at pseudo–critical values of the control parameter $c_2^*$ as a function of the system’s size $N_e$. As $N_e$ increases, $\langle s\rangle$ peaks become sharper and $c_2^*$ decreases as $c_2^* \sim 1/N_e$ (not shown), approaching the bulk critical point. Moreover, the magnitude of $\langle s \rangle |_{max}$ (i.e., its value at $c_2 = c_2^*$) follows a power-law with exponent $\gamma / \nu d \approx 0.7\,\pm \,0.01$ (Fig. 4B). To further determine the finite-size behavior of the system, we studied the cluster size distribution at the critical point. Figure 4C shows the CCDF of cluster sizes at the pseudo–percolation threshold for different values of $N_e$. At variance with the expectations for a percolation based model, namely a power-law with an exponential cutoff for large sizes, the CCDF for the AB model is consistent with cluster size distribution with *two* cutoffs

8 $$\begin{aligned} N_c(s) \sim \theta (s - s_0)\, s^{-(\tau - 1)} \ e^{-s/s^*}, \end{aligned}$$

where $\tau$ corresponds to Fisher exponent, the term $e^{-s/s^*}$ corresponds to the exponential cutoff for large sizes with $s^* \propto N_2$ and $s_0$ is a small-size cutoff (here $\theta (x)$ is the Heaviside step function, namely $\theta (x) = 1$ if $x > 0$ and $\theta (x) = 0$ otherwise). We see that, for intermediate an large scales, *n*(*s*) develops a power-law behavior with exponent $\tau = 2.38\,\pm \,0.04$ followed by a cutoff at size $s^*(N_e)$. As shown in the inset of Fig. 4C, $s^*$ and $s_0$ scale as a power-law with the system’s size with exponents $0.8\,\pm \,0.2$ and $0.5\,\pm \,0.1$, respectively. The cutoff $s^*$ is equivalent to $\langle N_2 \rangle |_{max}$, and therefore, both quantities are expected to scale as $N_{e}^{d_f/d}$. $\langle N_2 \rangle$ displays a growing maximum at $c_2^*$ that goes zero in the thermodynamic limit (not shown). Figure 4D shows the scaling of $\langle N_2 \rangle |_{max}$ as a function of $N_e$. In agreement with the exponent obtained for the scaling behavior of $s^*$, the power-law fitting of the data in Fig. 4D gives $d_f/d = 0.82\,\pm \,0.01$.

The presence of a small-size cutoff is a strong finite-size effect that disappears for large enough system sizes but has a strong impact on the estimation of the critical exponents at the scales of interest for mitochondria. For instance, for feasible simulation sizes, the estimated values of $\tau \approx 2.4$ and the cutoff exponent $d_f/d = 0.82\,\pm \,0.01$ are consistent with the standard percolation mean-field values $\tau =5/2=2.5$ and $d_f/d = 2/3 \approx 0.67$, an expected result in an infinite-dimension system^62^. However, the estimated value of the exponent $\gamma /\nu d=0.7\,\pm \,0.01$ (see Fig. 4B) is not consistent with the mean-field value $\gamma /\nu d=1/3$, which might be a direct consequence of the finite-size effect mentioned above.

### Critical exponents of the SE model are consistent with the 2D percolation universality class

The previous model recapitulates the universal dynamics found in many physical and biological systems near criticality without the need for any spatial information. Moreover, the AB model can produce networks that are structurally identical to real mitochondrial networks, raising the question of whether or not the spatial distribution of mitochondrial fragments within the cell is relevant for mitochondrial complexity^34^.

**Figure 5:**
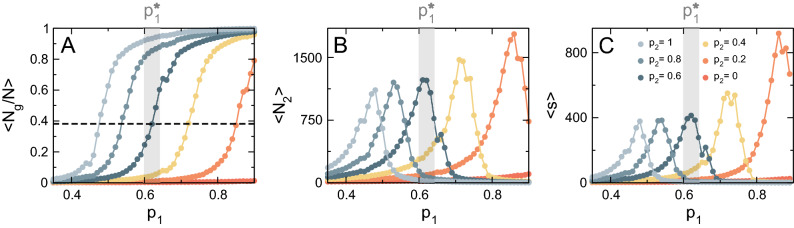
Phase transition in the SE model. (**A**) Order parameter $\langle N_g/N \rangle$ as a function of the control parameter $p_1$ for a system of size $N = 1 \times 10^4$ and different values of $p_2$. Shaded region depicts the pseudo–critical value $p^*_1$ for $p_2 = 0.6$. Dashed line denotes the proportion of nodes belonging to the largest cluster at the critical point for $p_2 = 0.6$. (**B**) Size of the second largest cluster $\langle N_2 \rangle$ as a function of $p_1$. (**C**) Susceptibility $\langle s \rangle$ as a function of $p_2$.

To answer this, we characterized the universal properties of the SE model, in which the association between mitochondrial units is constrained by their position in a 2D array (Fig. 2, bottom). As shown in Fig. 5A, the SE model exhibits a percolation-like phase transition as a function of $p_1$, evidenced by a sudden increase in the normalized size of the largest cluster at a pseudo–critical value $p^*_1$ (notice that $p_1$ is roughly equivalent to $c_1$ in the AB model). Accordingly, both $\langle N_2 \rangle$ and $\langle s \rangle$ peak at $p^*_1$, as shown in Fig. 5B,C (gray region is centered at $p^*_1$ for $p_2 = 0.6$ as reference).

**Figure 6:**
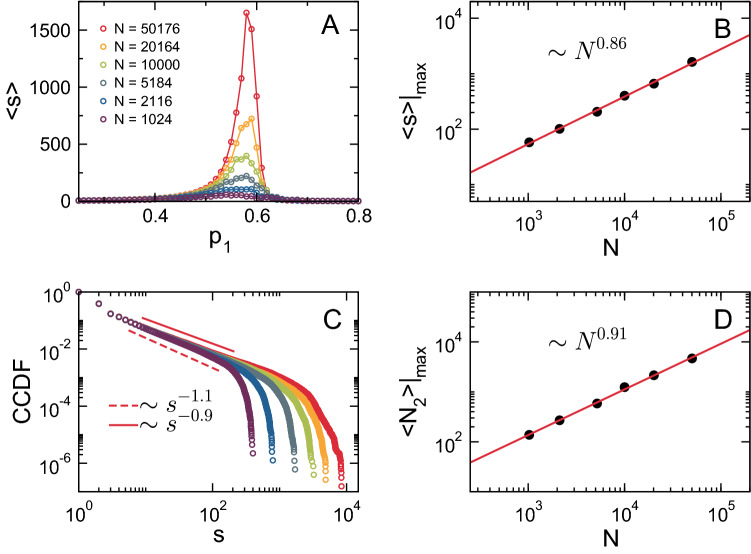
Finite-size scaling analysis of the SE model. (**A**) Susceptibility $\langle s \rangle$ as a function of $p_1$ for different system sizes ($p_2 = 0.7$). $\langle s \rangle$ exhibits a size-dependent maximum at a pseudo–critical value $p_1^*(N)$. (**B**) Log-log plot of $\langle s \rangle |_{max}$ as a function of *N*, where the solid red line is a power-law fitting with exponent $0.86\,\pm \,0.02$. (**C**) The CCDF of the cluster size distribution at $p_1^*(N)$ for different system sizes, where solid and dashed lines correspond to power-law fits of the central part of the distribution with exponents $-0.9\,(N = 50176)$ and $-1.1\,(N = 1024)$, respectively. (**D**) Log-log plot of $\langle N_2 \rangle |_{max}$ as a function of *N*, where the solid red line is a power-law fitting with exponent $0.91\,\pm \,0.02$.

We then studied the scaling behavior of relevant quantities as done for the AB model. Figure 6 illustrates the behavior of such quantities as we vary the system’s size. As shown in Fig. 6A, the system’s susceptibility exhibits a series of maxima at pseudo–critical values of the control parameter $p^*_1$ as a function of *N*. $\langle s \rangle$ peaks become sharper as *N* increases, following a power-law with exponent $\gamma /\nu d \approx 0.86\,\pm \,0.02$ (Fig. 6B). To further characterize the scaling behavior of the model, we computed the cumulative distribution function of cluster sizes at $p^*_1$ for different values of *N*. Figure 6C shows that the CCDF of cluster sizes exhibit the expected power-law behavior

9 $$\begin{aligned} N_c(s) \sim s^{-(\tau -1)} \ e^{-s/s^*}, \end{aligned}$$

where $\tau$ corresponds to Fisher exponent and the term $e^{-s/s^*}$ corresponds to a exponential cutoff at large cluster sizes with $s_* \propto N_2$. $n(s')$ develops a power-law behavior with exponent $\tau = 2.0\,\pm \,0.1$, followed by a cutoff at size $s^*(N)$. In agreement with the presence of a finite-size cutoff, $\langle N_2 \rangle |_{max}$ scales as function of *N* with exponent $d_f/d \approx 0.91\,\pm \,0.02$ (Fig. 6D).

In the case of the SE model, we can see that the estimated values of $\tau \approx 2.0$, $\gamma / \nu d = 0.86\,\pm \,0.02$ and $d_f/d = 0.91\,\pm \,0.02$ are all consistent with the 2D percolation critical exponents $\tau =187/91\approx 2.055$, $\gamma / \nu d =43/48\approx 0.896$ and $d_f/d =91/96 \approx 0.948$^62,69^.

### Critical exponents from MEFs’ mitochondrial networks are more consistent with the 2D percolation universality class

Our hypothesis implies that mitochondrial networks are dynamically tuned near the critical point of a phase transition. This is because mitochondrial mass fluctuates constantly, and as demonstrated in the previous sections, different system sizes require different pseudo–critical values of the control parameter to stay in the critical region^42^. We have shown that network quantities such as $\langle s \rangle$ and $\langle N_2 \rangle$ display size-dependent maxima at the critical point. Therefore, a testable prediction from our hypothesis is that the values of these network quantities computed from real mitochondrial networks of different sizes should behave similarly. In other words, if real mitochondrial networks are constantly tuned to their critical point, their network configurations should be equivalent to the ones found in the models at the critical point, and therefore, finite-size effects are expected^66^.

**Figure 7:**
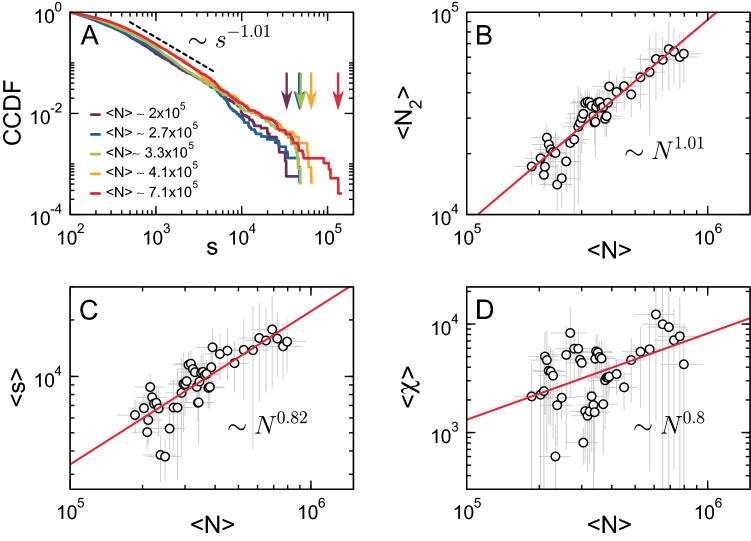
Finite-size scaling analysis of mitochondrial networks from MEFs. (**A**) The CCDF of the cluster mass distribution for different values of the average total mass of the network $\langle N \rangle$, where the dashed line corresponds to a power-law fit of the central part of the distribution with exponent $-1.01\,\pm \,0.01$. Arrows indicate how the large-size cutoff increases as a function of $\langle N \rangle$. (**B**) Log-log plot of $\langle N_2 \rangle$ as a function of $\langle N \rangle$, where the solid red line is a power-law fitting with exponent $1.01\,\pm \,0.06$. (**C**) Log-log plot of $\langle s \rangle$ as a function of $\langle N \rangle$, where the solid red line is a power-law fitting with exponent $0.82\,\pm \,0.08$. (**D**) Log-log plot of $\langle \chi \rangle$ (Eq. 10) as a function of $\langle N \rangle$, where the solid red line is a power-law fitting with exponent $0.8\,\pm \,0.23$. $\langle N \rangle$ corresponds to the average total mitochondrial mass estimated from images, used here as a proxy for network size. Symbols correspond to mean values and error bars to standard deviations from different intensity thresholds (see “Methods”).

In the following, we will use the mitochondrial mass (i.e., the number of pixels that show fluorescence) as a proxy of network size (*N*). We first studied the behavior of $n_s$ and its associated cutoff $s^*$ as a function of mass. Then, we ranked the networks based on their mass and divided them into five groups with increasing average mitochondrial mass $\langle N \rangle$. The CCDF of $n_s$ (Eq. 3) for each group of networks is shown in Fig. 7A, from which two different regimes can be distinguished: *i*) a power-law regime that spans almost two decades with exponent $\tau ' \approx 2$, and *ii*) a mass-dependent exponential cutoff (arrows), suggesting the presence of both scale invariance and finite-size effects.

Then, we investigated the behavior of $\langle N_2 \rangle$ as a function of the average mass $\langle N \rangle$. We sorted all networks from smallest to largest (mass) and applied a sliding window of size *n* to calculate the average mass of the second largest cluster and the average total mass of the networks. As shown in Fig. 7B and Supplementary Fig. 2, $\langle N_2 \rangle$ grows monotonically with the system’s size following a scaling law of the form $\langle N_2 \rangle \sim N^{\omega _1}$, where $\omega _1 = 1.01\,\pm \,0.06$.

We also computed $\langle s \rangle$ (Eq. 4) using the same sliding window approach. As shown in Fig. 7C and Supplementary Fig. 3, this quantity also follows a power law as a function of mass of the form $\langle s \rangle \sim N^{\omega _2}$, with $\omega _2 = 0.82\,\pm \,0.08$. As mentioned earlier, $\langle s \rangle$ is a proxy for the system’s susceptibility computed using the cluster size distribution^62^. Alternatively, the susceptibility can be computed from the fluctuations in the order parameter as

10 $$\begin{aligned} \chi = \langle N \rangle \left[ \bigg \langle \bigg (\frac{N_g}{N}\bigg )^2\bigg \rangle - \bigg \langle \frac{N_g}{N}\bigg \rangle ^2 \right] . \end{aligned}$$

Figure 7D and Supplementary Fig. 4 show that $\langle \chi \rangle \sim N^{\omega _3}$, with $\omega _3 = 0.8\,\pm \,0.23$, confirming our previous results. Note that, in agreement with theoretical expectations, both $\langle s \rangle$ and $\langle \chi \rangle$ seem to follow the same scaling law since $\omega _2 \approx \omega _3$.

In short, we have confirmed the presence of finite-size effects in real mitochondrial networks and obtained a set of exponents that inform on the underlying mitochondrial network dynamics. With some reservations, the following equivalencies can be established^62,68^

11 $$\begin{aligned} \tau '&\equiv \tau , \end{aligned}$$

12 $$\begin{aligned} \omega _1&\equiv \gamma / \nu d, \end{aligned}$$

13 $$\begin{aligned} \omega _2&\approx \omega _3 \equiv d_{f}/d, \end{aligned}$$

therefore allowing for a comparison with critical exponents from models and theory.

## Discussion

Results from simulations and experiments suggest that the networked structure of mitochondria emerges at the critical point of a percolation-like phase transition, where the *control parameter* is a function of fission and fusion rates^34,38^. Ideally, one should corroborate this by experimentally determining how the control parameter approaches the “bulk” critical point (i.e., the critical point for an infinitely large system) as the mitochondrial mass increases^42^. However, this would require imaging mitochondria at high spatial and temporal resolution for prolonged periods without causing significant cellular damage, something extremely challenging despite recent advances in super-resolution imaging techniques. Furthermore, the morphological features of the organelle make the identification of the smallest mitochondria constituents virtually impossible. Here, we circumvented this issue and determined near-criticality in mitochondrial dynamics by demonstrating the existence of finite-size effects in some relevant quantities of mitochondrial networks and identifying the associated critical exponents that describe the system’s behavior independently of its fine-grain details.

Using models, we illustrated how the complex structure of mitochondrial networks arises from the interaction between small mitochondria constituents at criticality. More precisely, a dynamic ensemble in the form of a networked structure composed of clusters, equivalent to the “default” mitochondrial network, emerges when the control parameter is tuned to its critical value (Figs. 3 and 5). If the value of the control parameter is set below the critical threshold, mitochondrial clusters remain segregated, and a giant cluster never reaches a critical mass to be stable. On the other hand, if the control parameter is set above the critical threshold, all mitochondrial clusters tend to merge into a single fully connected, more static network. In between these two extremes, we observe a dynamic regime that seems to be compatible with the structural properties found in real mitochondrial networks^34,38,40^.

Then, we showed that relevant network quantities become scale invariant at the critical point in both models by demonstrating that the magnitude of these quantities grows monotonically with the system’s size, as shown in Figs. 4 and 6. It is only at the critical point that this characteristic power-law behavior is observed since it stems from the divergence of the correlation length known to take place in the vicinity of a phase transition^42,62^. Although we found a similar scaling behavior in both models, the exponents of the power-laws, the critical exponents, were sufficiently different, indicating that they correspond to different universality classes.

Finally, we took advantage of the typically occurring fluctuations in mitochondrial mass and performed a finite-size scaling analysis in networks extracted from microscopy images of MEFs expressing a mitochondria-targeted fluorescent protein. Note that we use mitochondrial mass here as a proxy for the system’s size. Using a large set of high-resolution images, we covered a broad range of mitochondrial masses to quantify how network quantities behave as a function of mass. As shown in Fig. 7, these quantities scale with the mass following a power-law relationship, confirming that “healthy” mitochondrial dynamics lead to network configurations that are equivalent to the ones obtained from models at the critical point^34,38^.

Table 1 summarizes the critical exponents obtained from both models and mitochondrial images. Our results indicate that the AB model, besides some finite-size deviations, belongs to the *mean-field* universality class, while the SE model does so to the standard *2D percolation* universality class. Interestingly, though the AB model generates more “realistic” topologies compared to the SE model, the exponents obtained from real networks are more consistent with those of the 2D standard percolation universality class, suggesting that the spatial structure of the intracellular milieu might represent a constraint for the universal properties of these networks. Of note is that the critical exponents from real networks are much closer to the ones from 2D percolation than to the ones from 3D percolation, consistently with the idea that mitochondrial networks in “flat” cells are embedded in a 2-dimensional space.

Finally, our results support the idea that cells adjust mitochondrial fission and fusion (the control parameter) dynamically in response to mitochondrial mass fluctuations (the order parameter), similar to what has been reported in other biological systems^42,66^.

**Table 1:** Critical exponents determined using models and real mitochondrial networks. The exponents obtained using data from images of mitochondrial networks are more consistent with those of the 2D Percolation universality class. Theoretical exponents were extracted from Ref.^62^.

	$\tau$	${\gamma / \nu d}$	${d_{f}/d}$
Mean-Field Percolation	$5/2 = 2.5$	$1/3 \approx 0.33..$	$2/3 \approx 0.66..$
AB model	$2.38\,\pm \,0.04$	$0.70\,\pm \,0.01$	$0.82\,\pm \,0.01$
3D Percolation	2.15	0.67	0.84
2D Percolation	$187/91 \approx 2.055$	$43/48 \approx 0.896$	$91/96 \approx 0.948$
SE model	$2.0\,\pm \,0.1$	$0.86\,\pm \,0.02$	$0.91\,\pm \,0.02$
MEFs	$2.01\,\pm \,0.01$	$0.82\,\pm \,0.08$	$1.01\,\pm \,0.06$

## Methods

### AB model

The agent-based (AB) model follows closely the implementation described in Ref.^34^.

### SE model

We implemented the SE model on a square lattice of size $N = L^2$. With certain probability, two types of links are established: what we call “left/right links” and “side links”. In a two coordinates system, the right and left nearest neighbors of the *i*-est node (with coordinates (*i*, *j*)) would be nodes at positions $(i+1, j)$ and $(i-1, j)$, respectively (Fig. 2, bottom). Similarly, the side neighbor of the *i*-est node is the node located at position $(i, j\,\pm \,1)$. Notice that these definitions of left/right and side links are made only out of numerical and algorithmic convenience. Two parameters specify how links are established independently: $p_1$ is the probability for a node to be linked with both its left and right neighbors and $1 - p_1$ is the probability for the links between the *i*-est node and both its left and right neighbors to be destroyed (analogous to tip-to-side reactions in the AB model). In parallel, $p_2$ is the probability for a link between the *i*-est node and its side neighbor to be created and $1 - p_2$ is the probability for the link between the *i*-est node and its side neighbor to be destroyed (analogous to tip-to-side reactions in the AB model).

### Cell culture

We abide by the ARRIVE 2.0 guidelines^70^. Mice were housed in cages and had access to food and water ad lib at all times. Every effort was made to ameliorate animal suffering. All animal work was conducted in accordance with a protocol approved by the Institutional Animal Care and Use Committee at Weill Cornell Medical College. Mice used for experiments were between 8-14 weeks of age. Mouse embryonic fibroblasts (MEFs) were obtained as previously described^71^. Briefly, 13.5 days pregnant female C57BL/6 mice were sacrificed by $\hbox {CO}_{{2}}$ inhalation followed by cervical dislocation. Uterus was removed and embryos harvested, rinsed with PBS and placed on a petri dish. The head and red organs were discarded and the remaining tissue was chopped with razor blades and trypsinized for 15 min at $37{^\circ }$C. Trypsin reaction was quenched with Dulbecco’s Modified Eagle Medium (DMEM - GIBCO) supplemented with 10% FBS (GIBCO) and the mixture was centrifuged for 5 min at 2000 rpm. The cellular pellet was resuspended in DMEM supplemented with 10% FBS, 1% L-Glutamine (Sigma Aldrich), 1% Sodium Pyruvate (Sigma Aldrich), 1% HEPES (Sigma Aldrich) and 1% Penicillin/Streptomycin (GIBCO). Cell pellets from 4 embryos were seeded on 175 $\hbox {cm}^{2}$ culture bottles and allowed to grow for 48 h in a 5% $\hbox {CO}_2$ and $37{^\circ }\hbox {C}$ atmosphere.

Human Embryonic Kidney (HEK) 293T and Cos-7 cells (ATCC) were cultured in DMEM supplemented with 10% FBS, 1% L-Glutamine, 1% Sodium Pyruvate, 1% HEPES and 1% Penicillin/Streptomycin in a 5% $\hbox {CO}_2$ and $37{^\circ }\hbox {C}$ atmosphere. U2-OS cells (ATCC) were cultured in McCoy’s 5A Medium supplemented with 10% FBS and 1% Penicillin/Streptomycin in a 5% CO_2_ and 37^∘^C atmosphere.

### Plasmids, transfection, and lentiviral infection

The mitochondria-targeted YFP plasmid was purchased from OriGene. The ORF containing both the mitochondrial targeting sequence and the YFP was subcloned into the lentiviral plasmid pLV-eGFP to yield pLV-mitoYFP. pLV-eGFP (Addgene plasmid #36083; http://n2t.net/addgene:36083; RRID: Addgene_36083) and pLV-mitoDsRed (Addgene plasmid # 44386 ; http://n2t.net/addgene:44386; RRID:Addgene_44386) were a gift from Pantelis Tsoulfas. Human Tom20 cDNA (NM_014765) was cloned into a pAcGFP-N1 vector (Clontech) using NheI and AgeI restriction sites to produce Tom20-GFP. Cells were transfected with designated plasmids using Lipofectamine 2000 (Invitrogen) following the manufacturer’s instructions. Briefly, the transfection reaction was assembled in two tubes containing Opti-MEM (GIBCO), one with Lipofectamine and the other with the DNA mixture. After 5 min of incubation at room temperature, tubes were mixed and incubated for an additional 20 min at room temperature before being added to cells. Next, cells were incubated with the transfection mixture for 3 h, after which normal growth media was restituted. Lentiviral particles carrying the pLV-mitoYFP construct were produced as described before^72^. Briefly, HEK 293T cells were seeded onto 10 cm dishes, grown to ∼ 80% confluence, and co-transfected as described above with a polymerase-coding vector (pREV), a packaging vector (pRRE), an envelope vector (pVSV-G), and the shuttle vector carrying the sequence of interest. Media was collected at 48 h and 72 h, pooled and centrifuged for 5 min at 3000 g to pellet cell debris. The supernatant was aliquoted in 1.5 mL eppendorf tubes and spun down at 16000 g for 2 h at 4 °C to pellet lentiviral particles. The supernatant was discarded and dry lentiviral pellets were stored at − 80°C until use. Nuclei were visualized using DAPI (4′,6-diamidino-2-phenylindole) staining.

### Imaging

Images were collected on a Zeiss LSM 880 microscope equipped with the AiryScan detector using a 63x/1.4 NA Plan-Apochromat Oil DIC M27 objective lens (Zeiss) and an Edge 5.5 sCMOS camera (PCO). YFP was excited with a 488 nm Argon laser (5% power, $\sim 60 \mu W$) and collected with a 500/550 nm emission filter. Gain was set to 800. Scan mode was set to Frame with optimal frame size (3812x3812 pixels) resulting in an image pixel size of $\sim 35.29$ nm and a lateral resolution of $\sim 140$ nm. Speed was set to 8 ($2\mu s$ Pixel Dwell time) and the Bit depth at 16 bits. Prior to image analysis, raw *.czi* files were processed into deconvoluted Airyscan images using the Zen software with default settings.

### Image analysis

Data extraction from images was performed using a custom-written MATLAB code that extracts network quantities from *.czi* files produced by the ZEN software. The script first converts raw images to binary data by performing image thresholding. Subsequently, individual clusters are identified as groups of pixels connected by at least one of the eight nearest neighbors. These procedure yields a cluster distribution for each network analyzed from which all the relevant quantities used in this study can be obtained. Note that in this case the skeletonization step from Ref.^34^ was skipped to better estimate the mass of each cluster.

### Code availability

Codes and data are available at https://github.com/nahuelzamponi/mtmodels.

## Supplementary Information

Supplementary Information.
