# Supplementary material for: Universal dynamics of mitochondrial networks: a finite-size scaling analysis
Source: Sci Rep. 2022 Oct 12;12:17074. doi: 10.1038/s41598-022-14946-9 (PMC9556628; doi:10.1038/s41598-022-14946-9)
Supplement: Supplementary file 1 — Supplementary Information. [file 41598_2022_14946_MOESM1_ESM.pdf]

Universal dynamics of mitochondrial networks – A finite-size scaling analysis (Supplementary Material)

Nahuel Zamponi, Emiliano Zamponi, Sergio A. Cannas, Dante R. Chialvo

April 29, 2022

Two-dimensional representations of mitochondrial networks from "flat" cells constitute good approximations of their real structure

Although embedded in a volume, mitochondrial networks from MEFs (and most "flat" cells) are virtually spread on a two-dimensional space. To demonstrate this, we reconstructeded the three-dimensional structure of mitochondrial networks from 3 different types of flat cells (namely, MEFs, U2-OS, and Cos-7) by optical sectioning and measuring the X, Y, and Z axis dimensions of the embedding volume. As illustrated in Supplementary Figure 1, the length of the Z axis is minimal in comparison with the lengths of X and Y axes. In fact, except for a small region of the network near the nucleus, where cells are less "flat", the length of the Z axis is consistent with the diameter of one mitochondrial fragment (i.e., $\approx 0.5 - 1\,\mu m$[1, 2], lower panels in Supp. Fig. 1).

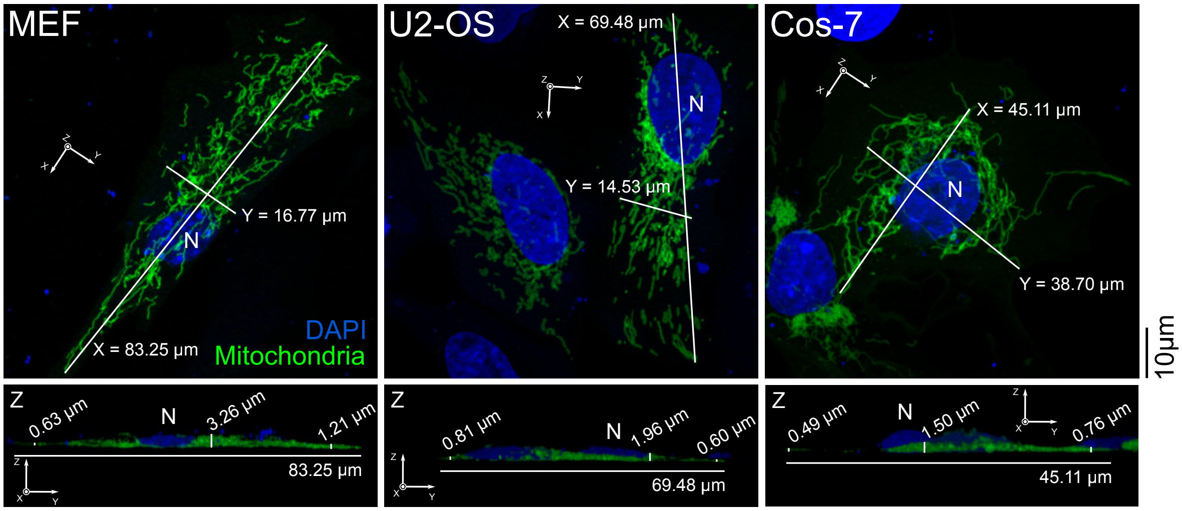

Supplementary Figure 1: Mitochondrial networks from "flat" cells. **Top panels**: measurements of the X and Y axes of mitochondrial networks from confocal images. **Bottom panels**: measurements of the Z axis of the embedding volume from 3D reconstructions of mitochondrial networks.

Critical exponents from real mitochondrial networks as a function of intensity threshold and window size

For each confocal image analyzed, an intensity threshold is established to determine the true signal from the background. Such threshold varied from image to image and was selected manually. To demonstrate that our results were robust to threshold selection, we defined a Δth and used three different intensity threshold values per image: the manual threshold (th^*) and two additional thresholds ($th^* \pm \Delta th$). Supplementary Figures 2, 3 and 4 depict the results of our analysis using two values of Δth and different windows sizes.

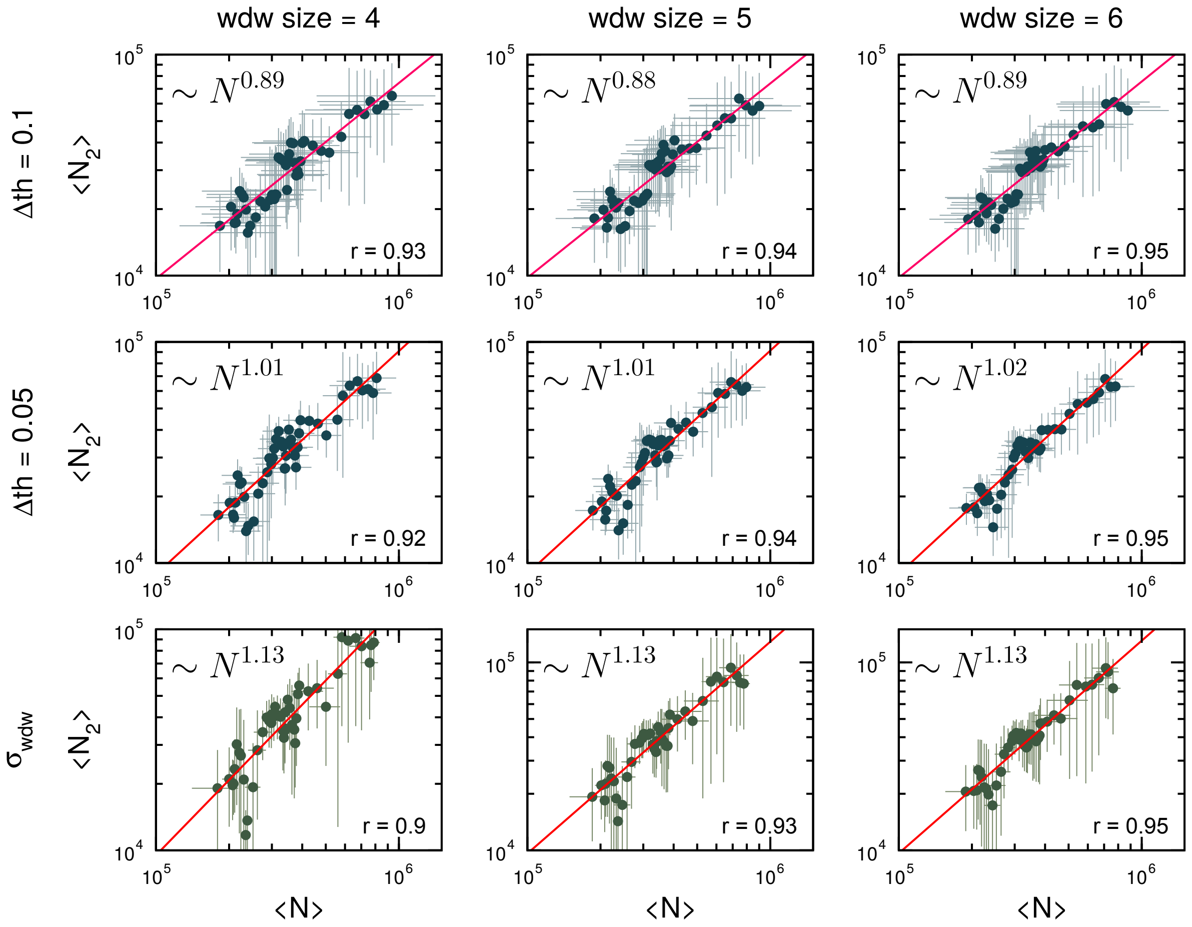

Supplementary Figure 2: Scaling of N_2 as a function of intensity threshold and window size. Columns correspond to different window sizes (4, 5 and 6). First two rows corresponds to different Δth values (0.1 and 0.05). Plots in the last row were computed using only the images from th^* (SD from images within each window).

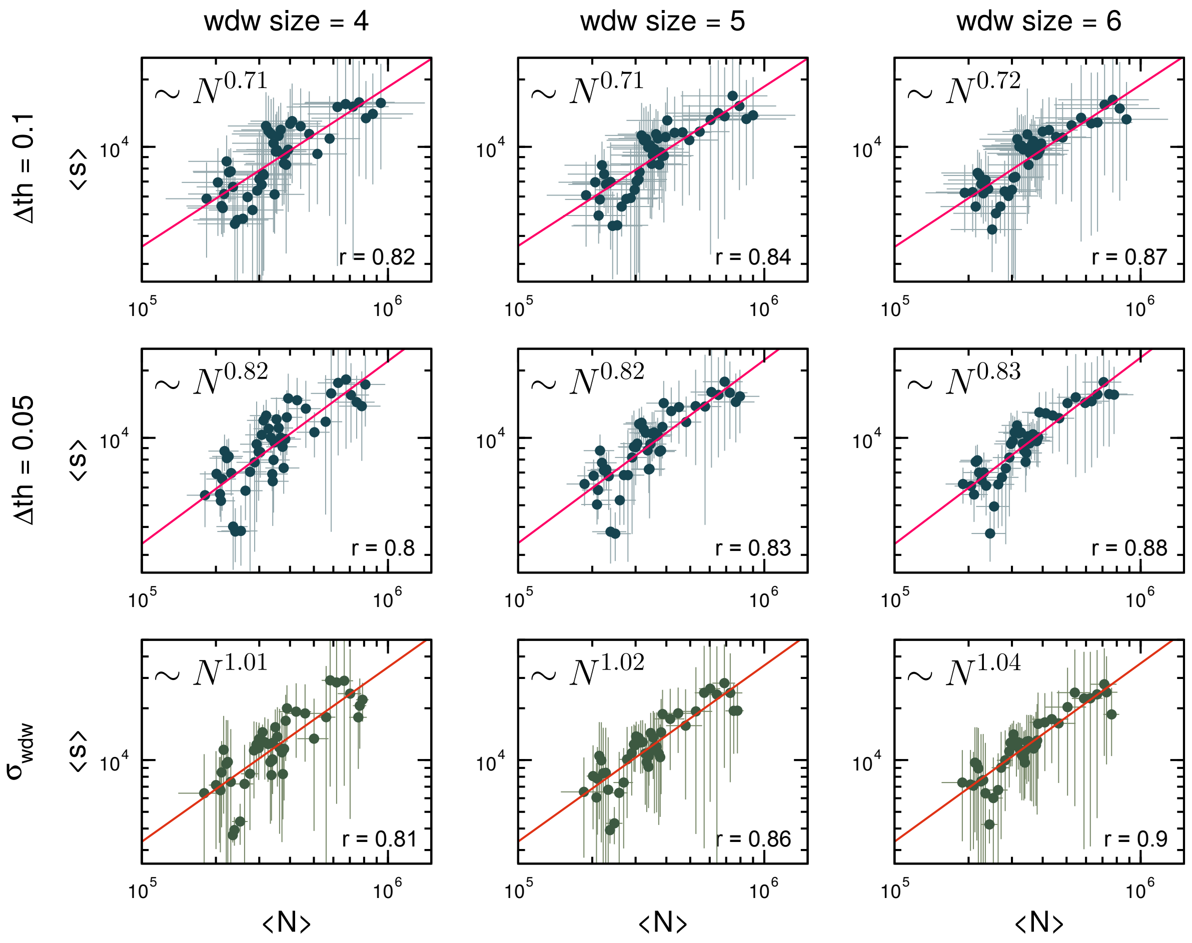

Supplementary Figure 3: Scaling of $\langle s \rangle$ as a function of intensity threshold and window size. Columns correspond to different window sizes (4, 5 and 6). First two rows corresponds to different Δth values (0.1 and 0.05). Plots in the last row were computed using only the images from th^* (SD from images within each window).

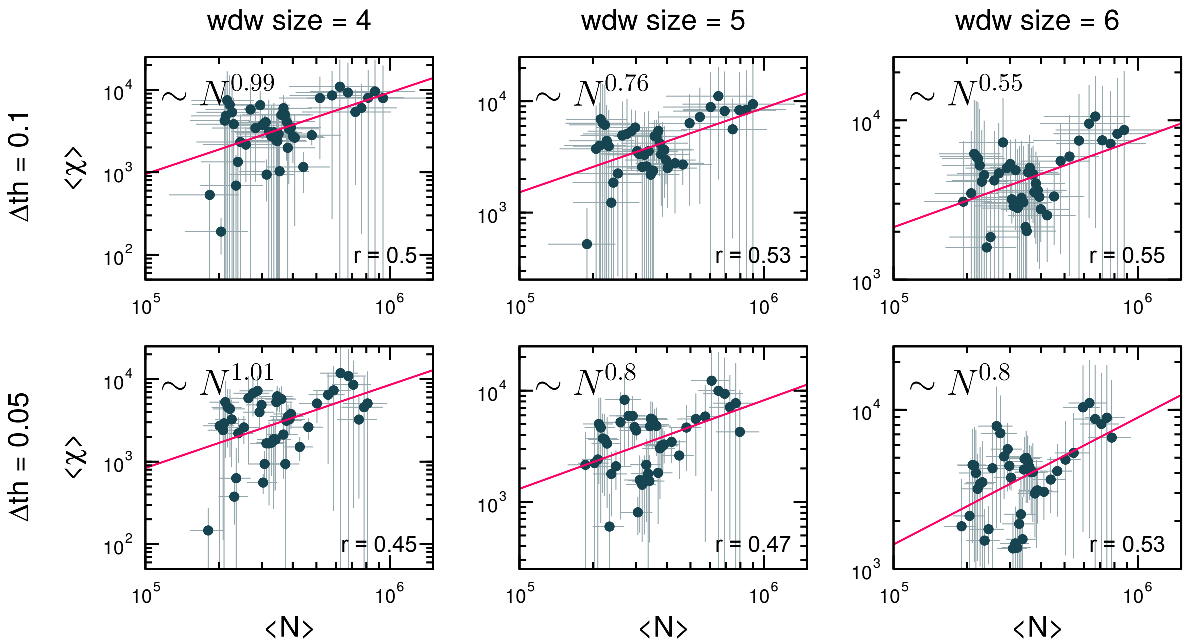

Supplementary Figure 4: Scaling of χ as a function of intensity threshold and window size. Columns correspond to different window sizes (4, 5 and 6). Rows corresponds to different Δth values (0.1 and 0.05).

References

[1] Long Q, Zhao D, Fan W, Yang L, Zhou Y, Qi J, Wang X, Liu X. Modeling of Mitochondrial Donut Formation. *Biophys J* **109**, 892-899 (2015). doi: 10.1016/j.bpj.2015.07.039

[2] Miyazono Y, Hirashima S, Ishihara N, Kusukawa J, Nakamura K-I, Ohta K. Uncoupled mitochondria quickly shorten along their long axis to form indented spheroids, instead of rings, in a fission-independent manner. *Sci Rep* **8**, 350 (2018). doi: 10.1038/s41598-017-18582-6
